# Comparative Evaluation of Color Stability and Fracture Resistance of CAD/CAM and Chairside Provisional Restorations: An In Vitro Study

**DOI:** 10.3390/jfb16110426

**Published:** 2025-11-20

**Authors:** Florina Titihazan, Ioana Veja, Cristian Zaharia, Tareq Hajaj, Cosmin Sinescu, George Dumitru Constantin, Mihai Rominu

**Affiliations:** 1 Department of Prostheses Technology and Dental Materials, Faculty of Dentistry, Victor Babes University of Medicine and Pharmacy, 2 Eftimie Murgu Sq., 300041 Timisoara, Romania; florina.titihazan@umft.ro (F.T.); sinescu.cosmin@umft.ro (C.S.); rominu.mihai@umft.ro (M.R.); 2 Research Center in Dental Medicine Using Conventional and Alternative Technologies, Faculty of Dental Medicine, Victor Babes University of Medicine and Pharmacy of Timisoara, 9 Revolutiei 1989 Ave, 300070 Timisoara, Romania; 3 Doctoral School, “Victor Babes” University of Medicine and Pharmacy Timisoara, Eftimie Murgu Square 2, 300041 Timisoara, Romania; george.constantin@umft.ro; 4 Department of Dental Medicine, Faculty of Dentistry, “Vasile Goldiș” Western University of Arad, 310025 Arad, Romania; veja.ioana@uvvg.ro; 5 Discipline of Clinical Skills, Department I Nursing, Victor Babeș University of Medicine and Pharmacy, 300041 Timisoara, Romania; 6 Center for Advanced Research in Cardiovascular Pathology and Hemostaseology, “Victor Babes” University of Medicine and Pharmacy Timisoara, 300041 Timisoara, Romania

**Keywords:** CAD/CAM, provisional crowns, color stability, fracture resistance, PMMA, high-impact polymer composite, digital prosthodontics

## Abstract

Background and Objectives: Provisional restorations are essential in fixed prosthodontics, ensuring esthetics, function, and biological protection during treatment. Recent advances in CAD/CAM technologies have enabled the fabrication of provisional materials with enhanced color stability and fracture resistance compared to conventional chairside polymeric materials. This study aimed to compare the color stability and fracture strength of provisional crowns fabricated using CAD/CAM and a conventional direct chairside technique. Materials and Methods: A total of 40 provisional crowns (four materials, *n* = 10 each group) were fabricated for a mandibular molar 3.6 using two workflows: CAD/CAM-milled poly(methyl methacrylate) (PMMA), high-impact polymer composite (HIPC; Bredent), and Ambarino composite (Creamet), and directly fabricated 3M™ Protemp™ (Scutan technique), respectively. Color stability was evaluated after seven-day immersion in coffee and red wine at 37 °C using a spectrophotometer (CIE L*a*b* system). Fracture resistance (*F*_max_) was measured under axial load in a universal testing machine. Data were analyzed by one-way ANOVA and Tukey’s HSD (*α* = 0.05). Results: Significant differences were observed among materials (*F*(3,36) = 212.6, *p* < 0.001). HIPC showed the highest mean fracture resistance (2068.9 ± 104.0 N), followed by PMMA (1215.8 ± 61.4 N) and 3M™ Protemp™ (1183.4 ± 86.4 N), while Ambarino exhibited the lowest (555.4 ± 25.4 N). Regarding color stability, Ambarino demonstrated the smallest ΔE* (1.1 ± 0.2), followed by PMMA (2.0 ± 0.3), HIPC (2.8 ± 0.3), and Protemp™ (4.9 ± 0.4). Only Protemp™ exceeded the clinical perceptibility threshold (ΔE* > 3.3). Conclusions: Both manufacturing methods and material compositions significantly influence the optical and mechanical properties of provisional restorations. CAD/CAM-milled HIPC and PMMA provided superior fracture strength and clinically acceptable color stability, suggesting their suitability for long-term or high-load temporary crowns compared with chairside-fabricated composites, particularly in posterior regions.

## 1. Introduction

Provisional restorations play a critical role in fixed prosthodontics by maintaining biological protection, occlusal stability, and esthetics during the transition from tooth preparation to final prosthesis placement. In clinical practice, discoloration or fracture of provisional crowns is most problematic in esthetically demanding anterior cases and long-span posterior temporizations, where repeated failures compromise patient satisfaction and treatment efficiency. These limitations highlight the need for materials combining superior optical stability with sufficient mechanical durability.

Despite the widespread clinical use of chairside provisional materials, their performance remains limited by polymerization shrinkage, surface roughness, and susceptibility to discoloration and fracture under masticatory stress. Recent innovations have introduced nano-filled PMMA and fiber-reinforced composites, which exhibit improved mechanical strength and reduced wear while maintaining optical translucency. Moreover, additive manufacturing technologies such as stereolithography and digital light processing (DLP) are emerging as promising alternatives to conventional milling, allowing for rapid, customized fabrication with reduced material waste. Nevertheless, further comparative studies are required to validate the clinical reliability of these new-generation materials and to establish standardized protocols for their use in long-term temporization.

When accurately fabricated, provisional crowns preserve pulpal vitality, protect exposed dentin. They also sustain periodontal health, and prevent tooth migration while allowing evaluation of occlusion, phonetics, and esthetics before final restoration [1,2,3,4]. These temporary restorations also function as diagnostic tools that help refine tooth contours, gingival architecture, and patient-specific esthetic expectations, thereby enhancing the predictability of definitive outcomes [5,6,7].

The rapid evolution of digital dentistry has revolutionized restorative workflows. Computer-aided design and computer-aided manufacturing (CAD/CAM) systems enable standardized and reproducible fabrication of both definitive and provisional restorations through precise digital workflows [8,9,10]. Advances in intraoral scanning, virtual design, and subtractive or additive manufacturing have improved dimensional accuracy, marginal integrity, and surface quality compared with conventional chairside techniques [11,12,13]. CAD/CAM-milled materials-particularly poly(methyl methacrylate) (PMMA) and high-impact polymer composites (HIPC)-are industrially polymerized under controlled conditions, resulting in higher degrees of conversion, reduced porosity, and enhanced mechanical and optical properties compared with self-curing resins [14,15,16].

Nevertheless, direct chairside techniques such as the Scutan method remain widely used due to their immediacy, cost-effectiveness, and ease of intraoral adjustment. This silicone-matrix technique employs self-curing resins (e.g., 3M™ Protemp™) to fabricate restorations in a single session [17,18,19,20]. However, direct provisionals are susceptible to polymerization shrinkage, microvoids, heat generation, and residual monomers, which may compromise pulp vitality marginal adaptation, color stability, and mechanical integrity-especially during long-term temporization [21,22,23].

The long-term success of provisional restorations depends primarily on color stability and fracture resistance. Color stability determines esthetic longevity in an oral environment influenced by chromogenic foods, beverages, and hygiene agents [24,25,26,27]. Conversely, fracture resistance reflects a material’s ability to withstand masticatory and parafunctional forces without deformation or catastrophic failure [28,29,30,31]. Fracture resistance testing provides essential insight into the ability of provisional materials to withstand masticatory and parafunctional forces without catastrophic failure. This property directly influences the clinical longevity and reliability of temporary crowns, particularly in posterior regions subjected to high occlusal loads. Previous research has reported ΔE* values of approximately 2–4 for chairside resins and fracture loads between 800 and 1500 N, defining clinically relevant thresholds for acceptable esthetic and functional performance [32,33,34,35]. Previous research has reported ΔE* values of approximately 2–4 for chairside resins and fracture loads between 800 and 1500 N, defining clinically relevant thresholds influenced by factors such as material composition, occlusal thickness, and abutment support conditions.

Given the increasing integration of CAD/CAM workflows in prosthodontic practice, comparing their performance with traditional chairside fabrication remains clinically important. In this study, three CAD/CAM-milled materials-PMMA, HIPC (Bredent), and Ambarino composite (Creamet)-were selected to represent distinct microstructural compositions and polymer matrices. Although Ambarino is less commonly used in daily prosthodontic practice, it was included for its innovative nano-hybrid formulation and enhanced esthetic properties, which justify evaluation alongside more established CAD/CAM materials. The direct Scutan-fabricated 3M™ Protemp™ served as a conventional control representing chairside techniques.

The selected CAD/CAM materials differ in chemical composition and polymer structure. PMMA is a highly polymerized poly(methyl methacrylate) resin, HIPC is a ceramic-reinforced polymer composite containing micro- and nano-fillers within a cross-linked methacrylate matrix, and Ambarino is a nano-hybrid resin composite with silanized glass fillers embedded in a polymer-infiltrated network. These compositional differences influence their optical behavior, degree of conversion, and resistance to fracture.

Therefore, this study aimed to compare the color stability and fracture resistance of provisional crowns fabricated using CAD/CAM milling and the Scutan direct technique. CAD/CAM milling and direct chairside fabrication were selected as the most commonly used techniques for provisional restorations in daily clinical practice, representing standardized digital and conventional workflows. Three-dimensional (3D) printing was not included because, despite its growing popularity, current printable resins show variable polymerization depth, limited mechanical strength, and insufficient long-term data compared to milled and self-cured materials. Future studies will extend the comparison to include 3D-printed provisionals as the technology continues to mature.

In this study, provisional crowns were fabricated for a mandibular first molar (tooth 3.6) abutment and cemented using TempBond NE (Kerr, Brea, California, USA). The null hypothesis stated that there would be no significant differences among materials in color change (ΔE*) or fracture load (*F*_max_). The null hypothesis stated that there would be no statistically significant differences among the tested materials in terms of color change (ΔE*) or fracture load (*F*_max_). By employing standardized test conditions in accordance with recommendations, this study seeks to provide evidence-based guidance for selecting provisional materials optimized for both esthetic and functional performance in modern prosthodontics [5,7,27,31].

## 2. Materials and Methods

### 2.1. Study Design and Objectives

This in vitro experimental study aimed to compare the color stability and fracture resistance of provisional crowns fabricated using two manufacturing techniques: computer-aided design/computer-aided manufacturing (CAD/CAM) milling and the direct Scutan chairside method.

A total of 40 provisional crowns were fabricated for a mandibular first molar (tooth 3.6) using four materials (*n* = 10 per group): poly(methyl methacrylate) (PMMA), high-impact polymer composite (HIPC; Bredent, Senden, Germany), Ambarino composite (Eckental, Germany), and 3M™ Protemp™ (Scutan technique; 3M ESPE, St. Paul, MN, USA).

The standardized abutment model reproduced a full-coverage tooth preparation with a 1.0 mm deep circumferential chamfer finish line, a 6° total occlusal convergence, and an occlusal reduction of approximately 1.5 mm. The axial wall height was set at 4 mm, providing uniform support for the provisional restorations. The study design adhered to relevant recommendations regarding specimen dimensions, loading configuration, and testing environment. Specifically, the minimum occlusal thickness (1.5 mm), crosshead speed (1 mm/min), and environmental conditioning before testing were selected in accordance with these standards to ensure methodological consistency and reproducibility. All provisional crowns were designed with a standardized occlusal thickness of approximately 1.5 mm and a minimum axial wall thickness of 1.0 mm. The preparation design simulated the clinical situation of a definitive full-contour monolithic crown (zirconia or lithium disilicate) intended for adhesive cementation using resin-based luting agents.

Two independent analyses were performed: (1) evaluation of color stability following immersion in staining solutions and (2) assessment of fracture resistance under axial loading until failure.

### 2.2. Model Fabrication and Digital Workflow

#### 2.2.1. Intraoral Scanning and Model Preparation

**Figure 1:**
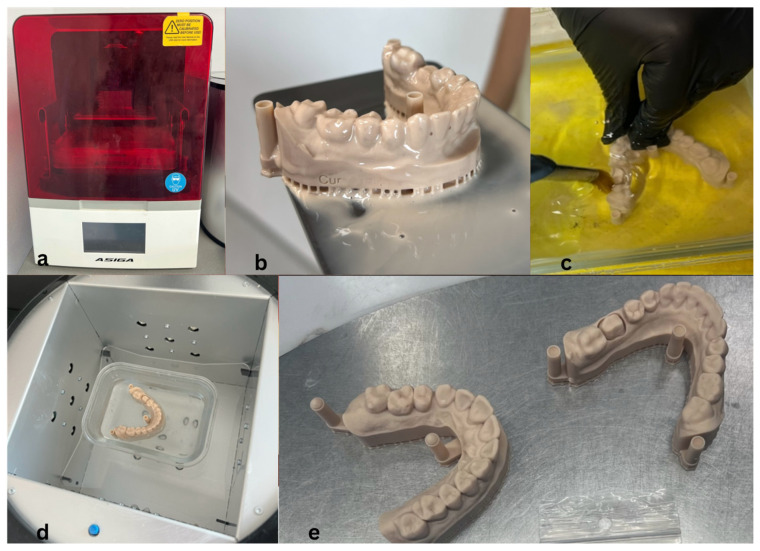
Three-dimensional printing process for master models: (**a**) Asiga MAX 3D printer; (**b**) printed model before cleaning; (**c**) IPA cleaning; (**d**) UV post-curing in glycerin; (**e**) final models.

Digital impressions of the maxillary and mandibular arches and occlusion were obtained using an intraoral scanner (Medit i700, Medit Corp., Seoul, South Korea). The scanner captures up to 70 frames per second, providing high-resolution three-dimensional surface data. The acquired scans were exported in STL format and processed in Asiga Composer v1.3.2; Asiga, Sydney, Australia software for virtual alignment and occlusal positioning prior to 3D printing (Figure 1a).

#### 2.2.2. Three-Dimensional Printing of Working Models

Working models were printed using an Asiga MAX 3D printer (Asiga, Sydney, Australia) with Asiga DentaMODEL resin (Figure 1b). The printer employs digital light processing (DLP) technology at a resolution of 10 µm, suitable for fixed prosthodontic applications. Following printing, models were cleaned twice in isopropyl alcohol (IPA), ultrasonically cleaned, and post-cured in glycerin under UV light to ensure complete polymerization and dimensional stability (Figure 1c–e). A single 3D-printed master model was used to fabricate all crowns, ensuring identical abutment geometry and standardized marginal adaptation across specimens, even without individual dies for proximal margin verification.

### 2.3. CAD Design and CAM Manufacturing

#### 2.3.1. Virtual Design and Articulation

**Figure 2:**
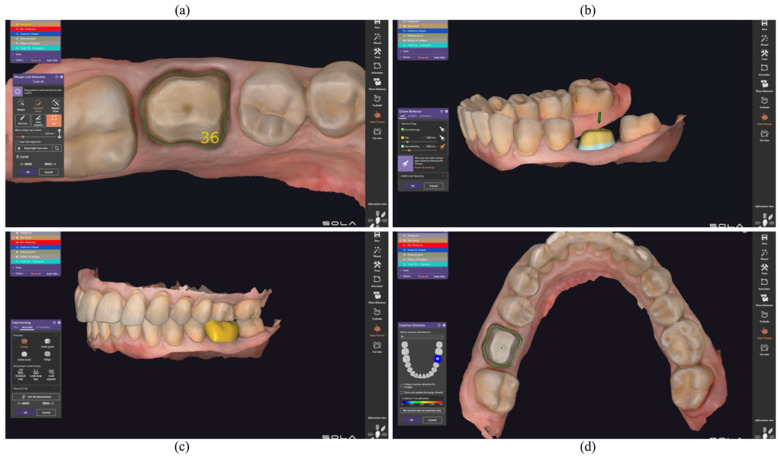
CAD design stages in Exocad: (**a**) defining preparation margin; (**b**) insertion axis; (**c**) final crown morphology; (**d**) cement space (60 µm).

The STL files of the printed models were imported into Exocad DentalCAD v3.1 Galway; Exocad GmbH, Darmstadt, Germany software (Exocad GmbH, Darmstadt, Germany) for virtual articulation and crown design (Figure 2). The workflow included defining the finish line (Figure 2a), establishing the insertion axis (Figure 2b), setting a standardized cement space of 60 µm (Figure 2c), and selecting crown morphology according to the occlusal scheme and restorative material (Figure 2d). The final designs of the temporary crowns were exported for milling.

#### 2.3.2. Milling Procedure

**Figure 3:**
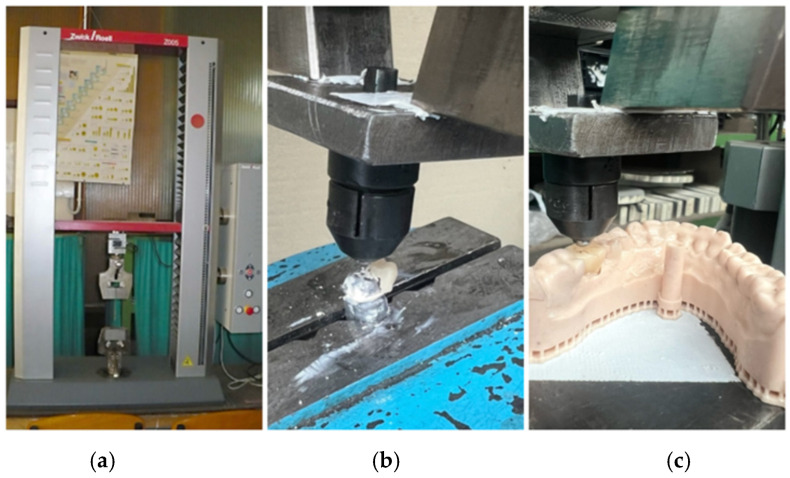
Fracture testing setup: (**a**) Zwick/Roell ProLine Z005 testing machine; (**b**) specimen fixation; (**c**) loading with hemispherical indenter.

All crowns were milled using a CORiTEC 650i five-axis wet milling system (imes-icore GmbH, Eiterfeld, Germany) to ensure high precision and surface finish (Figure 3a). The materials included PMMA disks, HIPC composite disks (Bredent GmbH & Co. KG, Senden, Germany), and Ambarino composite disks (Creamet, Eckental, Germany). Design files were optimized using iCAM V5 Smart software to achieve uniform nesting. After milling, crowns were separated from support pins, finished with rotary diamond instruments, and polished using diamond brushes and polishing paste until a glossy surface was obtained. Occlusal contacts were verified using articulating paper and adjusted, when necessary.

### 2.4. Fabrication of Provisional Crowns Using the Conventional Direct Chairside Technique

Ten provisional crowns were fabricated using the Scutan chairside method with 3M™ Protemp™ 4 Temporization Material (bis-acryl composite resin; 3M ESPE, St. Paul, MN, USA). The printed model was coated with a thin layer of petroleum jelly to prevent bonding. A prefabricated crown was used to create a silicone putty matrix, into which the Protemp™ material was injected using an automix dispensing gun. The matrix was reseated on the model until polymerization was complete. After removal, excess material was trimmed, minor defects were corrected with flowable composite, and the restorations were light-cured and polished to obtain a uniform, esthetic surface.

### 2.5. Experimental Evaluation

#### 2.5.1. Color Stability Assessment

All specimens were stored at 37 ± 1 °C in distilled water for 24 h to remove surface residues and residual monomers. Then, each specimen was then immersed in three media-coffee, red wine, and distilled water (control)-for seven consecutive days at 37 ± 1 °C. Solutions were renewed daily to prevent pigment saturation and simulate oral exposure.

Color measurements were performed before and after immersion using a VITA Easyshade V spectrophotometer (VITA Zahnfabrik, Bad Säckingen, Germany) calibrated to the VITA Classical shade guide and operating under the CIE L*a*b* color system. Three readings were averaged per specimen. For color stability testing, anatomically shaped full-contour crowns were used instead of flat disks to better replicate clinical reflection and translucency. Each specimen reproduced a standardized mandibular first molar (tooth 3.6) preparation with an occlusal thickness of approximately 1.5 mm, axial wall thickness of 1.0 mm, and total height of 4 mm, ensuring consistent geometry for spectrophotometric evaluation. Different baseline shades were selected according to the manufacturer’s standard availability for each material to ensure comparable optical thickness and translucency parameters within clinically relevant ranges. The chosen shades (PMMA-Bleach 1, HIPC-A2, Ambarino-B3, and Protemp™-A3) correspond to the lightest options routinely used in provisional restorations and were standardized to an initial L* value above 80 to minimize perceptual bias in color change measurements.

Color variation was calculated according to the CIE ΔE*_ab_ formula:

$$\Delta E_{ab}^{*}=\sqrt{(L_{2}^{*}-L_{1}^{*}{)}^{2}+(a_{2}^{*}-a_{1}^{*}{)}^{2}+(b_{2}^{*}-b_{1}^{*}{)}^{2}}$$

where L* denotes lightness, a* the red–green axis, and b* the yellow–blue axis.

A ΔE* value below 3.3 was considered clinically acceptable.

For PMMA specimens (Bleach 1 shade), measurements approached the upper detection limit of the spectrophotometer (L* > 95), slightly reducing quantitative accuracy. Although ΔE* could not be accurately quantified for PMMA (Bleach 1), the absence of detectable shade alteration suggests high stability within the instrument’s measurement limits.

Samples stored in distilled water exhibited negligible color variation (ΔE* < 1.0), confirming baseline stability of the materials and reliability of the measurement protocol.

#### 2.5.2. Fracture Resistance Testing

Each crown was cemented on a standardized metallic abutment replicating tooth 3.6 using TempBond NE (Kerr, Orange, CA, USA). Metallic abutments were selected after pilot tests with resin models led to premature failure, ensuring uniform stress distribution.

After 24 h of storage in distilled water at 37 °C, specimens were tested using a Zwick/Roell ProLine Z005 universal testing machine (Ulm, Germany, Figure 3a). A vertical compressive load was applied at the center of the distal occlusal fossa using a hemispherical steel indenter (4 mm diameter) at a crosshead speed of 1 mm/min until failure occurred. The compressive load was applied to the center of the distal occlusal fossa to reproduce the most common clinical contact area of mandibular first molars under centric occlusion. This point represents the primary load-bearing site where functional cusps typically engage during mastication, providing a realistic distribution of occlusal stress and better simulating clinical loading conditions. Force–displacement curves were recorded with TestXpert III software v1.71, and the maximum load at fracture (*F*_max_) was used for analysis. Fracture modes were visually classified as cohesive, adhesive, or mixed (Figure 3b,c).

Each crown was cemented onto custom-fabricated metallic abutments replicating the standardized digital design of tooth 3.6. The abutments were metallic, based on the same CAD model used for designing the crowns, ensuring geometric uniformity across all specimens. Preliminary testing on printed resin abutments revealed early substrate deformation; therefore, metallic replicas were used in the final experiments to provide consistent stress distribution and reliable load transfer during axial loading (Figure 3a–c).

### 2.6. Statistical Analysis

Statistical analysis was performed using SPSS v26.0 (IBM Corp., Armonk, NY, USA). Data normality was confirmed using the Shapiro–Wilk test (*p* > 0.05), and Levene’s test verified homogeneity of variances (*p* > 0.05).

Intergroup differences were analyzed by one-way ANOVA, followed by Tukey’s HSD post hoc test, and independent-samples t-tests were applied for selected pairwise comparisons. The significance level was set at *α* = 0.05.

An a priori power analysis (G*Power 3.1; effect size *f* = 0.35, α = 0.05, power = 0.80) indicated that 10 specimens per group (N = 40) were sufficient to detect clinically meaningful differences. A post hoc analysis confirmed observed power > 0.90 for both ΔE* and *F*_max_ comparisons.

## 3. Results

### 3.1. Color Stability

All specimens were conditioned in distilled water for 24 h to eliminate surface residues and residual monomers before testing. Each sample was subsequently immersed in two chromogenic solutions—coffee and red wine—for seven consecutive days at 37 °C, with daily renewal to simulate oral exposure. Color measurements were obtained using a VITA Easyshade V spectrophotometer (VITA Zahnfabrik, Bad Säckingen, Germany), calibrated to the VITA Classical shade guide and operating under the CIE Lab* color system. Color measurements were performed on anatomically shaped provisional crowns rather than flat specimens to better simulate clinical conditions, including light reflection and surface curvature. Although the CIEDE2000 (ΔE00) system offers improved perceptual uniformity, the traditional CIE Lab* (ΔEab) formula was used to ensure comparability with previous studies, which still references the ΔEab standard.

As shown in Table 1, the Ambarino composite (Creamet) maintained its original B3 shade, exhibiting the lowest color variation (ΔE* = 1.1 ± 0.2). The HIPC composite (Bredent) showed moderate color change (ΔE* = 2.8 ± 0.3), while the 3M™ Protemp™ crowns fabricated via the Scutan technique demonstrated the highest ΔE* values (4.9 ± 0.4), indicating statistically greater color alteration compared with the other groups (*p* < 0.05).

Quantitative analysis of ΔE* values using one-way ANOVA revealed statistically significant differences among the four materials (*F*(3,36) = 128.4, *p* < 0.001). Tukey’s HSD post hoc test identified Ambarino as significantly more color-stable than HIPC and Protemp™ (*p* < 0.05), while HIPC and PMMA did not differ significantly (*p* > 0.05).

For PMMA specimens (Bleach 1 shade), spectrophotometer readings approached the upper detection limit (L* > 95), which prevented reliable quantitative assessment. Consequently, these data were excluded from the ANOVA. Visual examination confirmed no observable discoloration, suggesting high color stability within the measurement limits of the device. The ΔE* value for PMMA (2.0 ± 0.3) was calculated from averaged spectrophotometric readings obtained within the upper measurement range of the device but excluded from ANOVA due to limited quantitative reliability near the detection threshold (L* > 95). Consequently, this value should be interpreted descriptively rather than statistically. Future studies will employ alternative calibration settings or spectrophotometers with extended luminance capacity to obtain fully comparable color data across all high-brightness materials.

Samples immersed in distilled water (control) showed negligible color variation (ΔE* < 1.0), confirming baseline stability.

Δ*E* value for PMMA (2.0 ± 0.3) was calculated from averaged spectrophotometric readings near the upper measurement range of the device (L* > 95). Due to limited quantitative reliability for high-luminosity materials, PMMA data were excluded from ANOVA statistical comparison.*

### 3.2. Fracture Resistance

All crowns were cemented onto standardized metallic abutments using TempBond NE (Kerr, Orange, CA, USA). Preliminary tests performed on 3D-printed resin abutments revealed early deformation and fracture of the resin substrate during loading, as evidenced by irregular force–displacement curves and visible cracking at low loads. Therefore, metallic abutments replicating the same CAD geometry were employed in the final testing phase to ensure uniform stress distribution and accurate load transfer (Figure 3a–c). All groups passed normality (Shapiro–Wilk, *p* > 0.05) and homogeneity (Levene, *p* > 0.05) tests.

**Figure 4:**
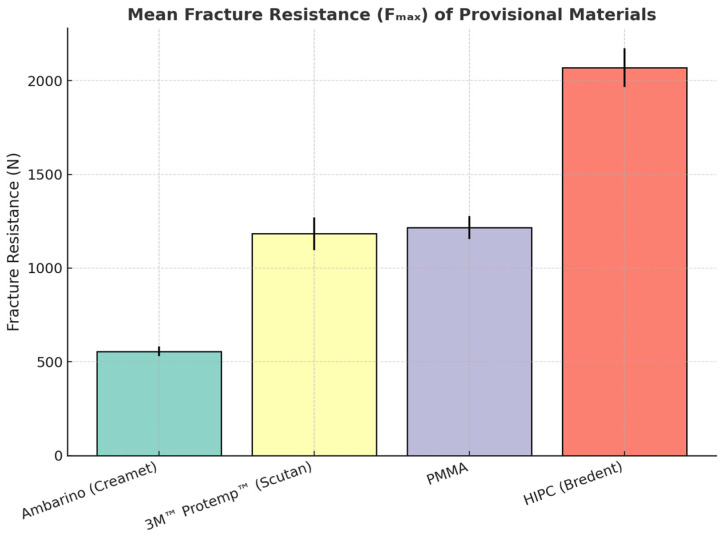
Mean fracture resistance (*F*_max_ ± SD) of provisional restorative materials fabricated by different techniques.

A vertical compressive load was applied to the occlusal surface at the center of the distal fossa, as this region provides a broad, stable contact area and represents a typical functional loading point in posterior occlusion. Loading at this position allows for more uniform stress distribution across the crown and avoids stress concentration on the cusp tips, which could otherwise lead to premature or non-representative fractures. The maximum load at failure (*F*_max_) was recorded using TestXpert III software v1.71, and the corresponding force–displacement curves were analyzed (Figure 4).

Descriptive and inferential statistics are summarized in Table 2. The highest mean fracture resistance was obtained for HIPC (2068.9 ± 104.0 N), followed by PMMA (1215.8 ± 61.4 N) and 3M™ Protemp™ (1183.4 ± 86.4 N), which were statistically similar. The Ambarino composite showed the lowest mean resistance (555.4 ± 25.4 N).

A one-way ANOVA revealed significant differences among the materials (F(3,36) = 212.6, *p* < 0.001). Tukey’s HSD test indicated four statistically distinct groups:

HIPC (a) > PMMA (c) ≈ Protemp™ (c) > Ambarino (d) (*p* < 0.05).

Pairwise *t*-tests were additionally performed to validate the direction and magnitude of intergroup differences, confirming the same hierarchy observed with Tukey analysis (Table 3). The use of *t*-tests provided complementary verification of pairwise contrasts and consistency across statistical methods.

Different superscript letters indicate statistically significant differences between groups (*p* < 0.05, one-way ANOVA with Tukey HSD post hoc test).

Failure analysis revealed material-dependent fracture patterns. HIPC crowns predominantly exhibited cohesive fractures with two major fragments, indicating high internal strength. PMMA and Protemp™ specimens mostly fractured into two or three large segments, consistent with brittle failure under compression. In contrast, Ambarino crowns showed multiple fragmentations and surface crumbling, suggesting lower structural integrity and crack resistance.

Fracture mode analysis showed that HIPC specimens predominantly exhibited cohesive failures (80%) with limited surface chipping, while PMMA and Protemp™ demonstrated mainly mixed fractures (70% and 60%, respectively). Ambarino crowns presented mostly adhesive failures (75%) accompanied by multiple fragmentations. All evaluations were performed independently by two calibrated examiners under blinded conditions, and discrepancies were resolved by consensus.

## 4. Discussion

The results of this study confirm that both the fabrication method and material composition play a decisive role in determining the optical and mechanical performance of provisional restorations. The observed ranking aligns with previous reports showing that CAD/CAM-milled polymers exhibit superior structural homogeneity, internal density, and reliability compared to conventionally fabricated, self-cured chairside resins [36,37,38,39,40,41,42,43,44]. This supports the growing evidence that industrial polymerization confers not only enhanced mechanical resistance but also more consistent esthetic outcomes.

From an optical standpoint, Ambarino (Creamet) demonstrated excellent color stability (ΔE* ≈ 1.1), followed by HIPC (Bredent), which presented a clinically acceptable shift (ΔE* ≈ 2.8). In contrast, 3M™ Protemp™ exhibited substantial discoloration (ΔE* ≈ 4.9), exceeding perceptibility thresholds. These variations reflect fundamental differences in polymer chemistry: cross-link density, filler morphology, and residual monomer content directly influence water sorption and pigment uptake [41,42,45,46]. HIPC’s moderate staining can be attributed to its ceramic-reinforced hybrid matrix, which enhances light diffusion and restricts chromogen penetration, whereas Protemp™ likely absorbed more pigments due to oxygen-inhibited surface layers typical of self-cured composites [46,47]. Although Ambarino exhibited the most favorable optical stability, its brittle structure and lower filler content resulted in reduced fracture resistance, limiting its suitability for provisional restorations in high-load posterior regions and indicating its potential use only for short-term applications under low masticatory stress.

In terms of mechanical behavior, a distinct hierarchy emerged: HIPC > PMMA ≈ Protemp™ > Ambarino. These findings are consistent with prior studies demonstrating that milled materials outperform chairside resins in fracture resistance due to improved polymer conversion and reduced internal voids [37,38,43,44]. The high load-bearing capacity of HIPC is likely related to its dense ceramic–polymer microstructure, which facilitates crack deflection and energy dissipation under compressive forces [44]. Meanwhile, PMMA and Protemp™ displayed similar short-term strengths, but PMMA’s industrial polymerization may yield better long-term fatigue resistance by minimizing residual stress and porosity [43,48]. The lower resistance of Ambarino likely results from its less cross-linked matrix and lower filler reinforcement, predisposing it to crack initiation and propagation under axial stress [43]. The predominance of cohesive fractures in HIPC suggests improved internal structural integrity, whereas mixed or adhesive failures in PMMA, Protemp™, and Ambarino indicate weaker interfacial bonding and lower crack resistance. These results align with prior findings that densely polymerized CAD/CAM materials better distribute stress within the bulk rather than at the cement interface. The higher fracture resistance observed for CAD/CAM-milled materials agrees with previous reports [], although some studies have reported comparable strength values for self-cured resins after mechanical aging [21]. These discrepancies may stem from differences in specimen geometry, curing kinetics, and loading configuration. HIPC’s superior performance can be attributed to its densely cross-linked ceramic–polymer microstructure, which enhances crack deflection and energy absorption under compressive stress. In contrast, the lower resistance of Ambarino likely results from reduced filler loading and lower cross-link density, predisposing it to microcracking.

The apparent inverse trend between optical stability and mechanical strength was interpreted qualitatively, as the present study did not include correlation analysis between ΔE* and *F*_max_. Future research should include statistical modeling to verify this relationship quantitatively.

The statement regarding oxygen-inhibited surface layers in Protemp™ is based on known polymerization behavior of bis-acryl resins reported in Doray et al. [19]; however, surface chemical analysis (e.g., FTIR or SEM–EDS) is recommended in future work to confirm this mechanism directly.

The standardized CAD design ensured uniform marginal and internal adaptation across specimens, minimizing variability in load distribution during testing. Precise adaptation likely contributed to the higher fracture load observed in CAD/CAM-milled groups by reducing stress concentration at the crown–abutment interface. Additionally, maintaining a consistent occlusal thickness of approximately 1.5 mm provided comparable structural support among materials, emphasizing that intrinsic material properties-rather than geometric factors-predominantly determined fracture resistance. While this explanation is consistent with known microstructural patterns, confirmation via scanning electron microscopy (SEM) or fractographic analysis is recommended for future work.

The apparent inverse trend between optical stability and mechanical strength-where the most esthetic material (Ambarino) was the weakest, and the strongest (HIPC) showed minor color change-should be interpreted qualitatively rather than as a verified correlation. This inverse relationship likely reflects fundamental material trade-offs: increasing filler loading and cross-link density enhances mechanical strength but reduces translucency and color stability due to greater light scattering and pigment accumulation at filler–matrix interfaces. Conversely, highly esthetic, less filled composites exhibit improved optical uniformity but lower resistance to fracture. The development of an ideal provisional material therefore remains challenging, requiring optimized nano-hybrid formulations that combine adequate filler reinforcement with controlled translucency to achieve both esthetic stability and mechanical durability.

This observation suggests that denser filler packing and higher cross-linking improve strength but reduce translucency and light transmission [43,48]. Clinically, this underscores the need for a tailored material selection: esthetic composites like Ambarino suit anterior temporization, while HIPC and PMMA are better adapted for posterior or long-term provisionalization requiring enhanced durability.

Beyond optical and mechanical properties, clinical workflow considerations should also be considered. Chairside techniques such as Scutan provide speed and cost efficiency-enabling same-day temporization-but are technique-sensitive and prone to variability. Conversely, CAD/CAM workflows require digital infrastructure and laboratory support but deliver reproducible precision, surface quality, and longevity. From a cost–benefit perspective, chairside techniques such as the direct method offer lower initial expenses and faster delivery but may require more frequent replacements due to staining or fracture. In contrast, CAD/CAM fabrication involves higher upfront costs and laboratory support yet provides improved longevity, reduced remakes, and greater standardization over time. Therefore, clinical decision-making should balance immediate affordability with long-term durability and patient satisfaction.

This study’s limitations include the absence of thermocycling, pH cycling, and mechanical fatigue, which restricts extrapolation to long-term intraoral performance. Additionally, surface roughness, gloss, and topographic parameters were not quantified, though they directly influence color stability. Testing was limited to uniaxial compressive loading, without simulating lateral or cyclic masticatory forces. The staining protocol employed distilled water, coffee, and wine solutions but excluded the effects of human saliva, temperature changes, or enzymatic degradation, which would better represent clinical aging.

Natural dental abutments were not used in order to eliminate biological variability such as differences in dentin hardness, moisture content, and morphology, which could have affected standardization and data reproducibility. Root morphology was intentionally excluded to eliminate anatomical variability and ensure standardized geometry across all samples. Metallic abutments with identical digital designs were used to provide uniform load transfer and minimize confounding factors. Only vertical loading was applied to isolate intrinsic material behavior under controlled conditions; however, future studies will include oblique loading and finite element stress analysis to evaluate the influence of complex masticatory forces and cement layer thickness on stress distribution and marginal adaptation. Instead, uniform metallic abutments were employed to ensure consistent geometry, homogeneous load distribution, and reliable comparison of material performance.

No thermocycling, pH cycling, or mechanical fatigue was applied in this preliminary experiment. This decision was made to isolate intrinsic material properties before introducing aging variables. The absence of aging procedures limits long-term extrapolation, which will be addressed in future ISO 4049- and 6872-compliant studies. The exclusion of PMMA color data due to spectrophotometer detection limits represents a major limitation, as it restricts full quantitative comparison among groups. Although visual inspection confirmed minimal perceptible discoloration, future studies should employ spectrophotometers with extended luminance range or alternative optical analysis methods to ensure accurate color assessment for high-brightness materials. This study was designed as a preliminary investigation to isolate and compare the intrinsic optical and mechanical properties of different provisional materials under standardized conditions. Aging and debonding simulations were intentionally excluded to avoid introducing additional variables, but future research will incorporate thermocycling, mechanical fatigue, and retention testing to better replicate long-term clinical performance.

Future research should therefore integrate multi-factorial aging models (thermal, pH, and cyclic loading) and surface characterization techniques (SEM, profilometry, gloss, and contact angle analyses) to elucidate the interplay between microstructure, Δ*E*, and fracture behavior* [43,46,48]. A schematic representation of the optical–mechanical balance (ΔE* vs. *F*_max_ correlation) would further illustrate how composition and processing affect performance.

The discussion on long-term performance refers to extended temporization cases, such as long-span provisionals or delayed definitive restorations, where enhanced durability is clinically relevant. The present findings are material- and brand-specific, and extrapolation to other provisional systems or fabrication techniques should be made with caution.

Within these limitations, the present findings reinforce the advantages of digital fabrication in modern prosthodontics. CAD/CAM-milled materials, particularly HIPC and PMMA, combine superior strength, reproducibility, and clinically acceptable esthetics, while the Scutan technique remains a practical, low-cost option for immediate or short-term provisional restorations.

## 5. Conclusions

Within the limitations of this in vitro study, both manufacturing technique and material composition significantly influence the optical and mechanical performance of provisional restorations. The superior fracture resistance of CAD/CAM-milled HIPC and PMMA may be partly attributed to their precise marginal adaptation and uniform occlusal thickness achieved through digital fabrication, which ensure optimal stress distribution during function. CAD/CAM-milled HIPC and PMMA demonstrated higher fracture resistance than directly fabricated composites while maintaining acceptable color stability. Although Ambarino exhibited the best color stability, its limited strength suggests use primarily for short-term use in low-load clinical situations. Protemp™ (Scutan), while practical and economical, showed higher staining susceptibility and lower mechanical resistance. Overall, CAD/CAM workflows provide a consistent, durable, and clinically reliable solution for provisionalization in contemporary single crown replacement prosthodontics. Future clinical trials should validate these in vitro trends under functional loading conditions.

## Figures and Tables

**Table 1:** Color variation (ΔE*) and shade changes in provisional crowns after immersion in staining solutions.

Material	*n*	Shade Before	Shade After	Δ*E* (Mean ± SD)
HIPC (Bredent)	10	A2	A3	2.8 ± 0.3
Ambarino (Creamet)	10	B3	B3	1.1 ± 0.2
3M™ Protemp™ (Scutan)	10	A3	A4	4.9 ± 0.4
PMMA	10	Bleach 1	Bleach 1	2.0 ± 0.3

**Table 2:** Mean fracture resistance (*F*_max_) and Tukey grouping of tested provisional materials (*n* = 10).

Material	Mean (N)	SD (N)	Tukey Group
Ambarino (Creamet)	555.41	25.40	d
3M™ Protemp™ (Scutan)	1183.40	86.40	c
PMMA	1215.78	61.36	c
HIPC (Bredent)	2068.92	103.99	a

**Table 3:** Pairwise independent-samples *t*-tests comparing fracture resistance (*F*_max_) among materials.

Comparison	Mean Difference (N)	Significance
HIPC vs. PMMA	853.1	***
HIPC vs. Protemp™	885.5	***
HIPC vs. Ambarino	1513.5	***
PMMA vs. Protemp™	32.4	ns
PMMA vs. Ambarino	660.4	***
Protemp™ vs. Ambarino	628.0	***

ns = not significant (*p* > 0.05); *** *p* < 0.001.

## Data Availability

The original contributions presented in the study are included in the article, further inquiries can be directed to the corresponding authors.
